# Efficacy and costs of combined balloon dilation plus atherectomy versus combined balloon dilation plus stent for lower-limb peripheral arterial disease: a single-center propensity-matched retrospective cohort study

**DOI:** 10.3389/fcvm.2026.1785484

**Published:** 2026-08-14

**Authors:** Haisheng Wu, Manrong Xu, Ruixin Wang, Yun Shen, Shengdi Lu, Ye Pan

**Affiliations:** 1 Department of Vascular Surgery, Shanghai Sixth People’s Hospital Affiliated to Shanghai Jiao Tong University School of Medicine, Shanghai, China; 2 Department of Endocrinology, Shanghai Sixth People’s Hospital Affiliated to Shanghai Jiao Tong University School of Medicine, Shanghai, China; 3 School of Public Health, Fudan University, Shanghai, China; 4 Division of Population and Public Health Science, Pennington Biomedical Research Center, Baton Rouge, LA, United States; 5 Department of Orthopaedics, Shanghai Sixth People’s Hospital Affiliated to Shanghai Jiao Tong University School of Medicine, Shanghai, China

**Keywords:** atherectomy, balloon angioplasty, cost analysis, drug-eluting stents, peripheral arterial disease, primary patency

## Abstract

**Objectives:**

To compare one-year vessel patency, limb perfusion and function, and direct and indirect costs between combined balloon angioplasty with rotational atherectomy versus balloon angioplasty with drug-eluting stent placement in patients with lower-limb peripheral arterial disease.

**Design:**

Single-center retrospective cohort study.

**Methods:**

Patients treated between January 2020 and December 2023 with atherectomy plus balloon angioplasty (AB group) were propensity-score-matched 1:1 to patients treated with balloon angioplasty plus a paclitaxel-eluting nitinol stent (BS group). The matched cohort included 160 patients per group. The primary outcome was 12-month primary vessel patency. Secondary outcomes were target vessel re-occlusion, ankle-brachial index (ABI), six-minute walk distance, Rutherford classification, disease-specific quality of life (Peripheral Artery Questionnaire, PAQ), major adverse cardiovascular events, and direct and indirect costs at one year.

**Results:**

In the matched cohort, 12-month primary patency was similarly high in both groups. Re-occlusion rates were comparable. The adjusted between-group ABI difference at 12 months was 0.010 (95% confidence interval −0.000 to 0.019; *p* = 0.060). The mean 6-minute walk distance improved by 121.1 m (AB) versus 122.5 m (BS) (*p* = 0.867). Rutherford category and PAQ score improved similarly in both groups (PAQ increase 25.6 vs. 26.2, *p* = 0.593). Major adverse cardiovascular events were rare [2 patients (1.3%) in each group by 3 months, none thereafter]. Mean total cost over one year was significantly lower in the AB group (93,189 CNY) than in the BS group (119,702 CNY; *p* < 0.001), driven by lower device/implant costs (27,574 vs. 54,536 CNY; *p* < 0.001).

**Conclusions:**

Atherectomy plus angioplasty was associated with 12-month patency and functional outcomes for which no significant difference from balloon angioplasty with drug-eluting stenting was detected, at significantly lower one-year cost. Because the design was powered for superiority rather than non-inferiority, these findings indicate absence of evidence of a difference rather than proven equivalence. The cost difference was driven almost entirely by the lower device price of the atherectomy-based strategy.

**Level of evidence:**

Therapeutic, Level III (retrospective matched cohort).

## Introduction

### Background

Lower-extremity peripheral artery disease (PAD) is an atherosclerotic occlusive disorder of the leg arteries that causes claudication and limb ischemia, and it ranks as the third leading cause of atherosclerotic cardiovascular morbidity after coronary and cerebrovascular disease (1). In 2019, an estimated 113 million adults aged 40 years and older worldwide were living with PAD (2). Symptomatic PAD impairs walking ability and increases the risk of limb loss and cardiovascular events, imposing a substantial public-health burden.

When PAD symptoms become lifestyle-limiting despite medical therapy, revascularization is indicated. Endovascular intervention is first-line for most femoropopliteal lesions, but plain balloon angioplasty alone has limited durability in long or heavily calcified lesions (3). Stenting improves primary patency relative to angioplasty alone, particularly in long or complex lesions; however, femoropopliteal stents remain susceptible to fracture and in-stent restenosis in the mobile leg arteries. These limitations have motivated adjunctive technologies, including drug-coated balloons, drug-eluting stents, and vessel-preparation strategies such as atherectomy (3).

Atherectomy mechanically debulks plaque before angioplasty. By excising calcific or fibrotic tissue, atherectomy may permit greater luminal gain with less elastic recoil, and randomized data indicate higher immediate technical success and fewer flow-limiting dissections or bailout stents than angioplasty alone (4). Long-term benefit, however, remains uncertain: a Cochrane review concluded that the evidence is of very low certainty and showed no clear improvement in 6- or 12-month patency or limb outcomes with atherectomy compared with plain angioplasty (5). In a recent real-world series of Jetstream rotational atherectomy for calcified femoropopliteal lesions, one-year primary patency was 88.9% with a 98.9% limb-salvage rate, although angiographically evident distal embolization occurred in 35.5% of cases and technical success was 93.4% (6). No published study has directly compared combined atherectomy plus balloon angioplasty against balloon angioplasty plus a contemporary drug-eluting peripheral stent on patency, limb function, patient-reported outcomes, and costs within a single propensity-matched cohort.

To address this gap, we performed a propensity-score-matched cohort study at Shanghai Sixth People's Hospital using data from January 2020 to December 2023, comparing atherectomy (Jetstream system) plus balloon angioplasty with balloon angioplasty plus paclitaxel-eluting nitinol stenting. We evaluated 12-month primary patency as the primary endpoint, alongside re-occlusion, ABI, walking distance, Rutherford category, patient-reported outcomes, and major adverse events, together with direct and indirect costs over one year.

## Methods

### Study design and setting

This retrospective single-center cohort study included patients treated at Shanghai Sixth People's Hospital from January 2020 through December 2023. Baseline demographic characteristics, general medical history, limb-specific history, and preoperative functional status were recorded for all participants. Propensity score matching was applied to balance baseline covariates and reduce confounding (7). The study design and reporting adhered to the STROCSS guidelines (8). The protocol was approved by the Ethics Committee of Shanghai Sixth People's Hospital [approval number 2024-KY-016(K)] and was conducted in accordance with the Declaration of Helsinki; no identifiable personal data were collected and individual informed consent was waived for this retrospective analysis.

### Participants

Adult patients (age >=18 years) with symptomatic lower-limb PAD treated at Shanghai Sixth People's Hospital between January 2020 and December 2023 were retrospectively screened through the hospital's Health Information System (HIS). Two treatment groups were eligible: patients treated with combined atherectomy plus balloon angioplasty (AB group) and patients treated with balloon angioplasty plus stent placement (BS group). PAD was defined as chronic atherosclerotic obstruction of the lower-limb arteries causing reduced blood flow, manifesting clinically as exertional thigh or calf pain (intermittent claudication), rest pain, or non-healing ulcers (9). Diagnosis required objective evidence of flow-limiting disease on noninvasive or angiographic testing (a resting ankle-brachial index <=0.90 or >=50% diameter stenosis on duplex ultrasound, computed tomography angiography, or digital subtraction angiography). The inclusion and exclusion criteria, which applied identically to both treatment groups, are shown in Table 1.

**Table 1:** Eligibility criteria.

Inclusion Criteria (applied identically to both groups)
1) Age >=18 years.
2) Documented symptomatic lower-limb PAD (as defined above) involving the femoropopliteal or infrapopliteal arteries, confirmed by duplex ultrasound, CT angiography, or digital subtraction angiography.
3) Treated at the study center during the enrollment period with either combined atherectomy plus balloon angioplasty (AB group) or balloon angioplasty plus stent placement (BS group) in the target limb.
4) At least 12 months of potential follow-up available from the date of the index procedure (i.e., index procedure performed on or before December 2022 was not required; all patients had a scheduled 12-month follow-up window within the study period).

Exclusion Criteria
1) Any prior revascularization (surgical bypass or endovascular intervention) in the same target limb.
2) Non-atherosclerotic arterial disease in the target limb (e.g., vasculitis, thromboangiitis obliterans).
3) Presentation with acute limb ischemia or embolic occlusion rather than chronic PAD.

Loss to follow-up was defined as failure to attend a scheduled follow-up visit (3, 6, or 12 months; +/- 2 weeks) with no outcome data recorded for that visit. The initial-treatment population comprised all 160 matched patients per group, analyzed according to their treatment group regardless of subsequent loss to follow-up. The as-treated population comprised patients who completed all scheduled follow-up assessments without protocol deviation. Patients lost to follow-up were retained in the initial-treatment primary-endpoint analysis using all available data under a mixed-model framework (described below); no outcome values were imputed.

### Intervention

All procedures were performed in a hybrid operating room under regional anesthesia (typically spinal or epidural) with conscious sedation. Under ultrasound guidance, antegrade common femoral arterial access was obtained and a 6–7 Fr sheath inserted. In the AB group, a 0.014-inch guidewire was advanced across the target lesion, and a Jetstream rotational atherectomy catheter (Boston Scientific, Marlborough, MA, USA) was used for vessel debulking under distal embolic protection (SpiderFX filter). Plain balloon angioplasty (sized 1:1 with the reference vessel) followed atherectomy, with inflation times of 2–3 min per lesion. In the BS group, the lesion was crossed with a guidewire and pre-dilated with a standard balloon, after which a self-expanding paclitaxel-eluting nitinol stent (Zilver PTX, Cook Medical, Bloomington, IN, USA) was deployed across the lesion, sized 1:1 to the vessel diameter, and post-dilated as needed. The detailed surgical protocols are provided in the Supplementary Materials.

### Postoperative care and rehabilitation protocol

After the intervention, arterial sheaths were removed and hemostasis achieved with manual compression or a femoral closure device. Patients received systemic heparin during the intervention to maintain an activated clotting time >=200–250 s, with protamine reversal if needed. Post-procedure, dual antiplatelet therapy (typically aspirin plus clopidogrel) was prescribed for 1–3 months, followed by indefinite single antiplatelet therapy. Early ambulation (after 4–6 h of bed rest) was encouraged. A standardized 12-week supervised exercise rehabilitation (walking) program was recommended in accordance with guidelines (Supplementary Materials).

### Outcome measures

#### Primary outcome

The primary outcome was 12-month primary vessel patency, defined by duplex ultrasound as freedom from >=50% diameter restenosis (peak systolic velocity ratio <2.5) without target-lesion reintervention (10). Patency was assessed by duplex ultrasound at routine 3-, 6-, and 12-month visits. The denominator for patency at each time point was the number of patients with a patency assessment available at that visit (the at-risk population); patients lost to follow-up before a given visit were not counted in that visit's denominator. At-risk counts at each time point are reported explicitly in Table 3.

#### Secondary outcomes

Secondary outcomes were assessed at 3, 6, and 12 months and included: (1) target vessel re-occlusion (total occlusion within the prior interval on duplex ultrasound); (2) the ankle-brachial index (ABI), measured as the ratio of the highest ankle systolic pressure to the highest brachial artery pressure (11); (3) the 6-minute walk distance (6MWT), a validated measure of functional walking endurance in PAD (12); (4) Rutherford ischemia category (0–6, from asymptomatic to major tissue loss) (13); (5) disease-specific quality of life measured by the 20-item Peripheral Artery Questionnaire (PAQ), a validated instrument whose summary score ranges from 0 to 100, with higher scores indicating better health status (fewer symptoms and better function); the PAQ assesses physical limitation, symptoms, social function, treatment satisfaction, and quality of life (14); and (6) major adverse cardiovascular events (MACE), defined as a composite of all-cause mortality, myocardial infarction, and stroke. A change in ABI of 0.15 was prespecified as the minimal clinically important difference (MCID) based on vascular-medicine consensus (11).

### Cost measures

Costs were assessed from a societal perspective over the 12-month horizon. Direct medical costs (hospital stay, primary and secondary outpatient care, physical therapy and rehabilitation, and implants/devices/medications) were extracted from hospital billing records. Direct non-medical costs (transportation for medical visits and specialized nutritional support) and indirect costs (productivity losses) were captured from patient-reported records collected at the 12-month outpatient visit. Indirect costs were estimated using the human-capital approach: patient and family-caregiver wage losses were calculated as days of work absence multiplied by the relevant documented or regionally imputed daily wage. All costs are reported in 2024 Chinese Yuan (CNY); costs incurred in earlier years were adjusted to 2024 price levels using the national consumer price index, and a 3% annual discount rate was applied to costs occurring beyond the first year (15).

### Adherence and adverse events

Adherence to the 12-week supervised rehabilitation program was assessed using four metrics retrieved from rehabilitation-department records and patient logbooks: completed daily exercise sessions (maximum 84), completed weekly self-examinations (maximum 12), follow-up phone calls received (maximum 4), and outpatient clinic visits attended (maximum 3). Adverse events (AEs) and serious adverse events (SAEs) were defined according to International Conference on Harmonisation Good Clinical Practice criteria and are detailed in Supplementary Table S4.

### Data collection

Data extraction was performed by two trained research assistants using a standardized electronic data-abstraction template. Two senior vascular surgeons independently verified eligibility and surgical details, and two senior physiotherapists reviewed rehabilitation records. None of the data abstractors or reviewers had provided clinical care to the included patients during the study period.

### Sample size calculation

Because this was a retrospective study, the sample size analysis confirmed that the available cohort had adequate statistical power to detect a clinically meaningful difference in the primary outcome. Using the prespecified ABI MCID of 0.15 and an assumed standard deviation of 0.15 in ABI change (11), a two-sided two-sample test with 80% power and alpha = 0.05 required 120 patients per group to detect a between-group difference of 0.15. After allowing for approximately 25% loss to follow-up, the target sample size was 160 patients per group, which the available cohort met. We emphasize that this calculation was powered to detect a superiority difference of one MCID; it was not designed as a non-inferiority study, and no non-inferiority margin was prespecified. The interpretation of non-significant between-group results is addressed accordingly in the Discussion.

### Data verification

To reduce analytical bias, the dataset was coded prior to analysis so that group labels (atherectomy plus angioplasty versus stenting plus angioplasty) and outcome identifiers were concealed from the primary statistician during initial data processing and modeling. Because this was a retrospective, non-randomized study, group concealment was implemented at the data-management stage: a data manager not involved in the analysis replaced the treatment-group variable with a neutral code before the dataset was released to the statistician, and the key was held separately until the primary models were fitted. An independent second analyst then verified key computations. This procedure represents independent data coding and analytical verification rather than formal prospective blinding, and we describe it as such.

### Propensity score matching

To reduce selection bias and confounding due to non-random treatment allocation, propensity score matching (PSM) was applied. The matching pool comprised all eligible patients treated at Shanghai Sixth People's Hospital between January 2020 and December 2023 who met the inclusion and exclusion criteria. Propensity scores were estimated using a multivariable logistic regression model that included baseline demographic data (age, sex), medical history (hypertension, diabetes, coronary artery disease, smoking status), limb-specific clinical data (Rutherford classification, lesion location and severity), and baseline functional status (ABI, walking distance, and PAQ score). One-to-one nearest-neighbor matching without replacement was performed with a caliper width of 0.2 standard deviations of the logit of the propensity score. Balance before and after matching was assessed using standardized mean differences (SMD), with a threshold of <0.10 indicating negligible imbalance; SMDs before and after matching are reported in Supplementary Table S5 and displayed graphically as a Love plot in Supplementary Figure S1.

### Statistical analysis

The distribution of continuous variables was examined using the Shapiro–Wilk test together with histograms and quantile-quantile plots. Approximately normally distributed continuous variables are presented as mean (standard deviation); cost variables, which were right-skewed, are additionally summarized as median (interquartile range, IQR). Categorical variables are presented as frequencies (percentages).

The primary analysis compared 12-month primary patency between groups in the initial-treatment population. Because patency is a binary outcome, between-group effects were estimated as risk differences and relative risks with 95% confidence intervals, and as odds ratios from a logistic regression model adjusted for baseline ABI; to account for the matched design, confidence intervals and tests used robust (cluster) standard errors clustered on the matched pair. Secondary continuous outcomes (ABI, 6MWT, PAQ) were analyzed using linear mixed-effects models with the outcome at 3, 6, and 12 months as the dependent variable. Fixed effects included treatment group, time (as a categorical factor), age, sex, and the baseline value of the outcome; a participant-level random intercept modeled within-subject correlation (compound-symmetry covariance), and a treatment-by-time interaction tested differential effects over time. Mixed models use all available observations and yield valid inference under a missing-at-random assumption when the predictors of missingness are included (16). Because missingness occurred only at the level of entire visits (never within a completed visit), no item-level imputation was required; as a sensitivity analysis, the mixed-effects results were compared with the complete-case (as-treated) analysis.

For cost outcomes, between-group differences in total and component costs were tested using both independent-samples t-tests and the nonparametric Mann–Whitney U test, with 95% confidence intervals for the difference in means obtained by nonparametric bootstrap (1,000 resamples) given the right-skewed distribution of costs (17). Because the two strategies yielded similar effectiveness while the atherectomy strategy was less costly, the economic comparison is framed in terms of dominance rather than per-unit incremental cost-effectiveness ratios; uncertainty is expressed using the bootstrap distribution of the cost difference and cost-effectiveness acceptability curves (Supplementary Table S3) (18). Subgroup analyses of the primary endpoint stratified by lesion location (femoropopliteal versus infrapopliteal) were prespecified as exploratory and are reported in Supplementary Table S6. All analyses were performed using R version 4.3 (R Foundation for Statistical Computing, Vienna, Austria); two-sided *p* < 0.05 defined statistical significance.

## Results

### Participants

**Figure 1:**
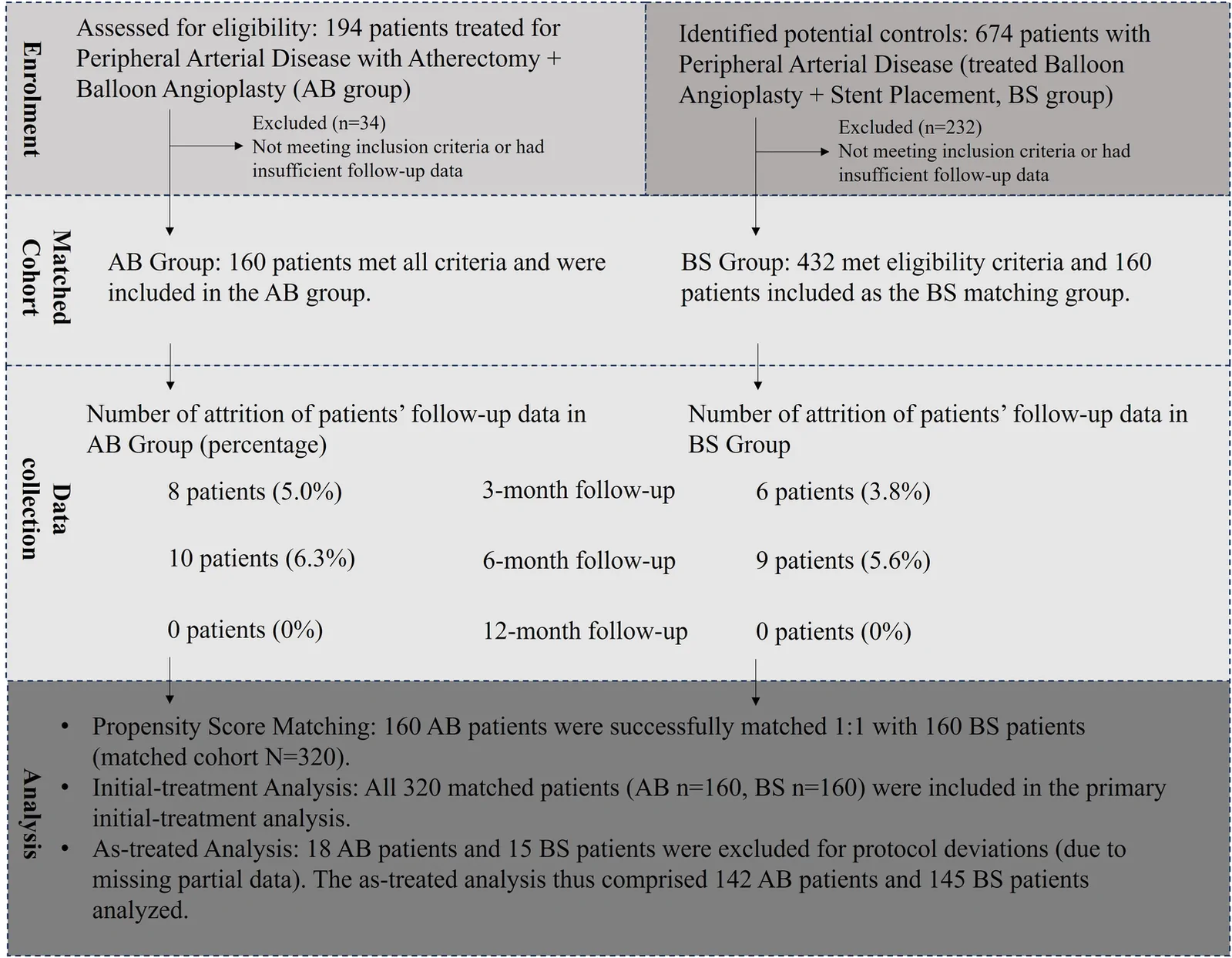
Flow diagram of patient screening, group allocation, follow-up attrition, and analysis populations for the AB and BS groups. A total of 194 patients treated with atherectomy plus balloon angioplasty (AB) were screened, with 34 exclusions, leaving 160 patients in the AB group. Of 674 patients treated with balloon angioplasty plus stent placement (BS), 232 were excluded, yielding 432 eligible patients; from these, 160 were selected by propensity score matching to form the BS group. During follow-up, 8 AB patients (5.0%) and 6 BS patients (3.8%) were lost by 3 months, with an additional 10 (6.3%) and 9 (5.6%) lost by 6 months, respectively; no further attrition occurred by 12 months. All 320 patients (160 per group) were included in the initial-treatment analysis. For the as-treated analysis, 18 AB and 15 BS patients were excluded due to protocol deviations, leaving 142 AB and 145 BS patients.

After screening 674 patients who received balloon angioplasty plus stent placement at the institution between January 2020 and December 2023, 432 met eligibility criteria and were included in the matching pool. Using 1:1 nearest-neighbor matching with a caliper of 0.2 standard deviations of the logit of the propensity score, 160 of these patients were matched to 160 patients treated with atherectomy plus balloon angioplasty (Figure 1). During follow-up, 8 patients (5.0%) in the AB group and 6 (3.8%) in the BS group were lost by 3 months, with an additional 10 (6.3%) and 9 (5.6%) lost by 6 months, respectively; no further attrition occurred by 12 months. All 320 matched patients (160 per group) were included in the initial-treatment analysis. For the per-protocol analysis, 18 AB and 15 BS patients were excluded for protocol deviations, leaving 142 AB and 145 BS patients.

After matching, baseline characteristics were well balanced between the AB and BS groups (Table 2). Standardized mean differences fell below 0.10 for all demographic, comorbidity, and lesion-related covariates after matching, compared with several imbalances before matching (Supplementary Table S5; Love plot, Supplementary Figure S1). The only residual difference was in baseline ABI, which was marginally higher in the AB group (0.73 +/- 0.06) than in the BS group (0.71 +/- 0.06; *p* = 0.017; SMD = 0.33); baseline ABI was therefore included as a covariate in all adjusted models. Baseline 6MWT, Rutherford classification, and PAQ scores were comparable. Sensitivity analysis in the per-protocol population (Supplementary Table S1) confirmed no significant baseline imbalances.

**Table 2:** Baseline characteristics of the matched cohort.

Initial-treatment	Characteristic	AB group (*N* = 160)	BS Group (*N* = 160)	P value
**Baseline Characteristics**	Male Gender [no. (%)]	91 (56.88)	89 (55.63)	0.822
	Age (yrs)	60 (4.99)	60 (5.54)	0.899
	BMI (kg/m^2)	23 (2.85)	23 (3.09)	0.738
	Occupation			0.877
	Manual worker	135 (84.38)	136 (85.00)	
	Non-manual worker	25 (15.63)	24 (15.00)	
	Education level			0.818
	Lower than high school	63 (39.38)	61 (38.13)	
	Equal/higher to high school	97 (60.63)	99 (61.88)	
	Insurance type			0.929
	Government	120 (75.00)	119 (74.38)	
	Commercial	3 (1.88)	4 (2.50)	
	Self	37 (23.13)	37 (23.13)	
	Current Smoker	56 (35.00)	54 (33.75)	0.814
	Current Alcohol use	56 (35.00)	58 (36.25)	0.815
	Paracetamol and NSAIDs	7 (4.38)	5 (3.13)	0.556
**Medical History**	Coronary Artery Disease	7 (4.38)	5 (3.13)	0.556
	Stroke/TIA or carotid stenosis	4 (2.50)	5 (3.13)	0.735
	Congestive heart failure	4 (2.50)	3 (1.88)	0.702
	COPD or asthma	5 (3.13)	6 (3.75)	0.759
	Chronic Kidney Disease	3 (1.88)	5 (3.13)	0.474
	Antiplatelet Therapy	136 (85.00)	133 (83.13)	0.647
	Statin Therapy	158 (98.75)	160 (100.00)	1.000
	PAD Symptom Duration (mo)	72 (7.69)	72 (7.48)	0.982
**Limb-specific Data**	Affected Limb Laterality			0.367
	Left	87 (54.38)	95 (59.38)	
	Right	73 (45.63)	65 (40.63)	
	Lesion Location			0.181
	Femoropopliteal	64 (40.00)	58 (36.25)	
	Infra-popliteal	96 (60.00)	102 (63.75)	
	Lesion Length			0.050
	focal <=1 cm	33 (20.63)	27 (16.88)	
	short >1-<5 cm	95 (59.38)	110 (68.75)	
	intermediate 5-<15 cm	31 (19.38)	18 (11.25)	
	long >=15 cm	1 (0.63)	5 (3.13)	
	Degree of Stenosis			0.690
	mild (<50%)	50 (31.25)	53 (33.13)	
	moderate (50–69%)	102 (63.75)	96 (60.00)	
	severe (70–99%)	8 (5.00)	11 (6.88)	
	Multi-level disease	19 (11.88)	24 (15.00)	0.412
**Function**	Ankle-Brachial Index (ABI)	0.73 (0.06)	0.71 (0.06)	0.017
	6-minute walk test (m)	241 (50.55)	240 (56.34)	0.862
	Rutherford classification			0.511
	Mild	12 (7.50)	11 (6.88)	
	Moderate	146 (91.25)	144 (90.00)	
	Severe	2 (1.25)	5 (3.13)	
	Peripheral Artery Questionnaire (PAQ)	53 (5.90)	52 (6.04)	0.519

AB: atherectomy + balloon angioplasty; BS: balloon angioplasty + stent placement; BMI: body mass index; NSAIDs: Non-Steroidal Anti-Inflammatory Drugs; TIA: transient ischemic attack; COPD: Chronic Obstructive Pulmonary Disease; PAD: Peripheral Arterial Disease. Values are mean (standard deviation) for age, BMI, PAD symptom duration, and functional measures (ABI, 6MWT, PAQ); others are number (percentage).

### Primary outcome

At 12 months, primary patency was present in 149 of 152 patients with an available assessment in the AB group (98.0%) and 150 of 153 patients in the BS group (98.0%) (Table 3). The at-risk denominators at each visit (152 and 153 at 12 months, after accounting for loss to follow-up) are reported explicitly in Table 3; percentages are calculated relative to the number of patients assessed at each time point rather than the full matched N of 160. The between-group risk difference at 12 months was 0.0% (95% CI −3.1% to 3.1%; relative risk 1.00, 95% CI 0.97–1.03), and the baseline-ABI-adjusted odds ratio with matched-pair robust standard errors showed no significant difference (*p* = 0.994). Per-protocol results were consistent (Supplementary Table S2).

**Table 3:** Outcomes at 3, 6, and 12 months in the matched cohort (intention-to-treat).

Outcome	3-month			6-month			12-month		
	AB group	BS Group	P value	AB group	BS Group	P value	AB group	BS Group	P value
Patients at risk (n)	153	156		152	153		152	153	
Primary Patency [n (%) of at-risk]	152 (99.35)	154 (98.72)	0.993	149 (98.03)	151 (98.69)	0.647	149 (98.03)	150 (98.04)	0.994
Re-occlusion (last 3 months)	1 (0.65)	1 (0.64)	0.993	2 (1.32)	1 (0.65)	0.558	0 (0.00)	1 (0.65)	1.000
ABI change from baseline	0.15 (0.09)	0.16 (0.08)	0.186	0.16 (0.10)	0.17 (0.10)	0.288	0.17 (0.10)	0.17 (0.10)	0.818
6MWT change from baseline (m)	104.39 (61.25)	108.61 (70.68)	0.576	115.76 (69.76)	116.01 (75.64)	0.976	121.07 (70.29)	122.52 (80.90)	0.867
Rutherford classification			0.270			1.000			0.867
Mild	140 (91.50)	133 (85.81)		142 (93.42)	143 (93.46)		145 (95.39)	144 (94.12)	
Moderate	12 (7.84)	21 (13.55)		8 (5.26)	8 (5.23)		6 (3.95)	8 (5.23)	
Severe	1 (0.65)	1 (0.65)		2 (1.32)	2 (1.31)		1 (0.66)	1 (0.65)	
PAQ change from baseline	17.65 (7.86)	18.32 (8.33)	0.465	23.05 (8.97)	23.01 (7.50)	0.972	25.62 (8.62)	26.15 (8.74)	0.593
MACE	2 (1.31)	2 (1.29)	0.990	0 (0.00)	0 (0.00)	1.000	0 (0.00)	0 (0.00)	1.000

AB: atherectomy + balloon angioplasty; BS: balloon angioplasty + stent placement; ABI: ankle-brachial index; 6MWT: 6-minute walk test; PAQ: peripheral artery questionnaire; MACE: major adverse cardiovascular events (composite of all-cause mortality, myocardial infarction, and stroke). Primary patency percentages are calculated relative to the number of patients with an available assessment at each visit (“patients at risk”); patients lost to follow-up before a given visit are excluded from that visit's denominator. ABI, 6MWT, and PAQ values are the mean change from baseline [mean (SD)]; positive values indicate improvement. P values compare AB versus BS at each time point; all values are unadjusted.

### Secondary outcomes

Both groups showed clinically meaningful improvement in ABI from baseline. The mean ABI change from baseline was 0.17 (SD 0.10) in the AB group and 0.17 (SD 0.10) in the BS group at 12 months, corresponding to post-procedure ABI values of approximately 0.90 and 0.88, respectively; both exceeded the prespecified MCID of 0.15. The adjusted between-group difference in ABI at 12 months was 0.010 (95% CI −0.000 to 0.019; *p* = 0.060), well below the MCID. For the 6-minute walk test, the mean improvement from baseline to 12 months was 121.07 m (AB) and 122.52 m (BS) (*p* = 0.867; adjusted between-group difference 0.237 m, 95% CI −8.898 to 9.372; *p* = 0.959). Rutherford classification improved similarly in both groups. The mean improvement in PAQ score at 12 months was 25.62 (AB) and 26.15 (BS) (*p* = 0.593; adjusted difference 0.004, 95% CI −0.654 to 0.661; *p* = 0.991). Target vessel re-occlusion was rare and did not differ between groups (Table 3). Unadjusted results are shown in Table 3 (and Supplementary Table S2 for the per-protocol population); adjusted results are shown in Table 4.

**Table 4:** Adjusted between-group effects at 3, 6, and 12 months.

Outcome	3-month			6-month			12-month		
	Coefficient	95% CI	P value	Coefficient	95% CI	P value	Coefficient	95% CI	P value
Initial-treatment population									
ABI	0.008	(−0.001, 0.018)	0.093	0.008	(−0.001, 0.017)	0.099	0.010	(−0.000, 0.019)	0.060
6MWT (m)	−1.707	(−8.918, 5.504)	0.643	−0.431	(−8.654, 7.792)	0.918	0.237	(−8.898, 9.372)	0.959
PAQ	−0.055	(−0.893, 0.784)	0.898	0.035	(−0.666, 0.735)	0.923	0.004	(−0.654, 0.661)	0.991
As-treated population									
ABI	0.007	(−0.002, 0.015)	0.143	0.006	(−0.004, 0.015)	0.235	0.008	(−0.003, 0.018)	0.160
6MWT (m)	−1.302	(−8.732, 6.129)	0.731	−0.326	(−8.882, 8.229)	0.940	1.120	(−8.500, 10.740)	0.820
PAQ	−0.063	(−0.923, 0.798)	0.887	0.088	(−0.619, 0.794)	0.808	0.037	(−0.631, 0.704)	0.915

ABI: Ankle-Brachial Index; 6MWT: 6-minute walk test; PAQ: Peripheral Artery Questionnaire. Each coefficient is the adjusted between-group difference in change from baseline (AB minus BS); positive values favor AB. Models were linear mixed-effects models adjusted for baseline value, age, and sex, with a participant-level random intercept and a treatment-by-time interaction.

### Safety

Major adverse cardiovascular events were rare, occurring in 2 of 160 patients (1.3%) in each group by 3 months, with no events at 6 or 12 months. Peri-procedural and other adverse events, including access-site complications, vessel perforation, flow-limiting dissection, and distal embolization, were uncommon and did not differ significantly between groups; detailed adverse events and serious adverse events are presented in Supplementary Table S4. Any adverse event occurred in 5 of 160 patients (3.1%) in the AB group versus 3 of 160 (1.9%) in the BS group, and serious adverse events occurred in 1 (0.6%) versus 2 (1.2%) patients, respectively, with no significant between-group difference.

### Cost outcomes

Mean total 12-month cost was significantly lower in the AB group (93,189 CNY; median 92,150, IQR 80,430–105,920) than in the BS group (119,702 CNY; median 118,640, IQR 106,880–132,410; *p* < 0.001 by both t-test and Mann–Whitney U test) (Table 5). The per-protocol analysis showed the same pattern (93,434 vs. 119,726 CNY; *p* < 0.001; Supplementary Table S3). Among individual cost components, only implant/device/medication cost differed significantly, being lower in the AB group (27,574 CNY vs. 54,536 CNY; *p* < 0.001). All other direct medical, direct non-medical, and indirect cost components showed no significant between-group difference (*p* > 0.05). The bootstrap 95% confidence interval for the total cost difference was −26,513 CNY (95% CI −30,940 to −22,090), excluding zero.

**Table 5:** 12-month costs in the matched cohort (intention-to-treat).

Cost (CNY)	AB group (*N*=160)	BS Group (*N*=160)	P value
Direct medical cost			
Hospital stays	6,997 (967.23)	6,981 (1,009.05)	0.884
Primary care	2,512 (454.98)	2,500 (511.58)	0.831
Secondary care	2,820 (3,102.42)	2,892 (3,188.95)	0.840
Physical therapist	10,327 (1,982.82)	10,091 (1,995.04)	0.301
Implant/Device/Medication	27,574 (2,478.51)	54,536 (4,519.52)	<0.001
Direct non-medical cost			
Transportation	1,984 (303.32)	2,003 (311.66)	0.598
Nutrition	4,448 (520.25)	4,491 (440.86)	0.438
Indirect cost			
Lost wages (patients)	32,587 (17,184.40)	32,376 (16,360.28)	0.912
Lost wages (families)	3,940 (4,625.48)	3,832 (4,215.11)	0.832
Total cost, mean (SD)	93,189 (18,391.53)	119,702 (18,213.17)	<0.001
Total cost, median (IQR)	92,150 (80,430–105,920)	118,640 (106,880–132,410)	<0.001

CNY: Chinese Yuan; AB: atherectomy + balloon angioplasty; BS: balloon angioplasty + stent placement; SD: standard deviation; IQR: interquartile range. Component costs are reported as mean (SD). Total cost is reported as both mean (SD) and median (IQR) because cost data were right-skewed; the between-group difference in total cost was significant by both the independent-samples t-test and the Mann–Whitney U test (*p* < 0.001), with a bootstrap 95% confidence interval for the difference in means of −30,940 to −22,090 CNY. All costs were captured from a societal perspective and are reported in 2024 CNY; earlier-year costs were adjusted to 2024 price levels using the consumer price index, and costs beyond the first year were discounted at 3% annually.

Because the two strategies produced similar effectiveness across all clinical measures (no between-group difference reached the prespecified MCID) while the atherectomy-based strategy was substantially less costly, the atherectomy-based strategy was economically dominant (similar effectiveness at lower cost). Accordingly, we do not report per-unit incremental cost-effectiveness ratios, which are uninformative and numerically unstable when the effectiveness difference is near zero and statistically non-significant; instead, uncertainty is summarized by the bootstrap distribution of the cost difference and by cost-effectiveness acceptability curves (Supplementary Table S3). At a willingness-to-pay of zero (cost-minimization), the probability that atherectomy was cost-saving while at least as effective exceeded 0.85 for all three effectiveness measures.

### Subgroup analysis by lesion location

In exploratory subgroup analyses stratified by lesion location (Supplementary Table S6), 12-month primary patency was similarly high for femoropopliteal and infrapopliteal lesions, with no significant between-group difference within either subgroup. These subgroup analyses were underpowered (femoropopliteal: 64 AB and 58 BS patients; infrapopliteal: 96 AB and 102 BS patients) and are reported as exploratory only; the findings should not be extrapolated to definitive lesion-specific conclusions.

### Adherence

Adherence to the rehabilitation program was comparable between groups (Table 6). The mean number of completed daily exercise sessions over the 12-week supervised program (maximum 84) was 63.3 (SD 14.2) in the AB group versus 62.4 (SD 14.8) in the BS group (*p* = 0.338); other adherence metrics were also similar.

**Table 6:** Adherence to the 12-week supervised rehabilitation program (intention-to-treat).

Adherence metric	AB group (*N* = 160)	BS Group (*N* = 160)	P value
Completed daily exercise sessions (out of 84)	63.3 (14.2)	62.4 (14.8)	0.338
Completed weekly self-examinations (out of 12)	9.1 (2.1)	8.9 (2.2)	0.338
Follow-up phone calls received (out of 4)	3.3 (0.6)	3.4 (0.7)	0.238
Outpatient clinic visits attended (out of 3)	2.7 (0.4)	2.7 (0.4)	1.000

AB: atherectomy + balloon angioplasty; BS: balloon angioplasty + stent placement. Values are mean (standard deviation). The maximum for each metric reflects the protocol-specified total over the 12-week program (84 daily sessions, 12 weekly self-examinations, 4 phone calls, 3 outpatient visits). Adherence data were retrieved from patients’ logbooks and the Health Information System.

## Discussion

In this propensity-matched cohort of patients with lower-limb PAD, atherectomy plus balloon angioplasty (AB) and balloon angioplasty plus a paclitaxel-eluting nitinol stent (BS) were associated with 12-month primary patency, functional outcomes, and safety for which no significant between-group difference was detected, while the AB strategy was associated with significantly lower one-year cost. Three points warrant emphasis. First, because the study was powered to detect a superiority difference of one ABI MCID and no non-inferiority margin was prespecified, the non-significant between-group results indicate absence of evidence of a difference rather than demonstrated equivalence (19). Second, the cost difference was driven almost entirely by the lower device price of the atherectomy-based strategy rather than by differences in the broader care pathway. Third, the favorable patency observed in both arms must be interpreted in light of the cohort's selection and the limitations described below.

### Interpretation in the context of femoropopliteal device evidence

Our findings should be interpreted against the peripheral (rather than coronary) endovascular literature, because the mechanical environment, stent platforms, restenosis mechanisms, and outcomes of femoropopliteal and infrapopliteal arteries differ substantially from those of the coronary circulation. In pivotal femoropopliteal trials, paclitaxel-eluting and paclitaxel-coated devices have improved patency relative to plain angioplasty or bare-metal stents: the Zilver PTX randomized trial reported 5-year primary patency of 66.4% versus 43.4% for standard care (20); the IN.PACT SFA trial reported 12-month primary patency of 82.2% versus 52.4% for a drug-coated balloon versus angioplasty (21); the LEVANT 2 trial reported 65.2% versus 52.6% (22); and the IMPERIAL trial demonstrated non-inferiority of a polymer-coated paclitaxel-eluting stent to Zilver PTX (12-month primary patency 86.8% versus 81.5%) (23). The high patency observed in our cohort is consistent with the upper range of these reports and likely reflects the predominance of short, non-occlusive lesions and the exclusion of patients with prior revascularization or critical limb ischemia.

### The paclitaxel mortality signal

Because the BS arm used a paclitaxel-eluting device, the late-mortality signal associated with paclitaxel-based peripheral devices must be acknowledged. A 2018 study-level meta-analysis of randomized trials reported increased all-cause mortality with paclitaxel-coated balloons and stents in the femoropopliteal artery at 2 years (7.2% versus 3.8%; risk ratio 1.68, 95% CI 1.15–2.47) and 5 years (14.7% versus 8.1%; risk ratio 1.93, 95% CI 1.27–2.93) (24). Although subsequent patient-level analyses and registry data have been less consistent, this signal remains clinically relevant and is a further consideration favoring a non-drug-eluting strategy where outcomes are otherwise comparable. Our 12-month follow-up is too short to assess late mortality, and this is an important limitation given the device used in the comparator arm.

### Femoropopliteal versus infrapopliteal disease

Our cohort combined femoropopliteal and infrapopliteal lesions, which differ in treatment strategy, expected patency, indications for stenting, and reintervention risk (25, 26). Infrapopliteal (below-the-knee) arteries exhibit more medial calcification and higher recoil, generally lower patency, and more restricted stenting indications than femoropopliteal arteries (27). Although exploratory subgroup analyses by lesion location showed no significant between-group differences, these analyses were underpowered, and our overall results should not be generalized to lesion-specific recommendations. This limitation is now stated explicitly.

### Economic interpretation

From a societal perspective, the 12-month cost was approximately 26,500 CNY lower with atherectomy, a difference driven by device/implant cost; hospital stay, rehabilitation utilization, transportation, and productivity losses did not differ. The economic advantage is therefore best characterized as device-price dominance under conditions of similar effectiveness, rather than a broader value advantage arising from differences in care or recovery. This framing avoids the well-recognized instability of per-unit incremental cost-effectiveness ratios when the effectiveness difference is near zero (18).

### Strengths and limitations

Strengths include propensity matching with formal balance diagnostics, baseline-adjusted mixed-effects modeling, and independent analytical verification. Several limitations temper the findings. The retrospective, single-center design permits residual confounding and selection bias despite matching. The exclusion of patients who completed fewer than 75% of rehabilitation sessions likely selected for healthier, more adherent patients and may have contributed to the high patency rates; this means the cost conclusion reflects a device-price difference within a favorable-risk population rather than a generalizable value advantage. Loss to follow-up produced whole-visit missing data, raising the possibility of attrition bias if dropouts differed systematically from completers. The 12-month horizon precludes assessment of late patency and of the paclitaxel mortality signal. Finally, the combination of lesion territories and the modest sample size limit subgroup inference. These factors reduce external validity and warrant cautious interpretation.

## Conclusion

In this propensity-matched cohort of patients with lower-limb PAD, atherectomy plus balloon angioplasty was associated with 12-month patency, functional recovery, and safety for which no significant difference from stent-based angioplasty was detected, at significantly lower one-year cost. Because the study was powered for superiority rather than non-inferiority, these results indicate absence of evidence of a difference rather than proven equivalence, and the cost advantage is attributable primarily to the lower device price of the atherectomy-based strategy. Prospective, adequately powered, multicenter studies with longer follow-up, prespecified non-inferiority margins, lesion-specific stratification, and preference-based utility measures are needed to confirm these findings.

## Data Availability

The raw data supporting the conclusions of this article will be made available by the authors, without undue reservation.
