# Supplementary material for: Efficacy and costs of combined balloon dilation plus atherectomy versus combined balloon dilation plus stent for lower-limb peripheral arterial disease: a single-center propensity-matched retrospective cohort study
Source: Front Cardiovasc Med. 2026 Aug 14;13:1785484. doi: 10.3389/fcvm.2026.1785484 (PMC13522772; doi:10.3389/fcvm.2026.1785484)
Supplement: Supplementary file 1 [file Datasheet1.docx]

**Supplementary Materials**

**Methods**

**Intervention**

All interventions were conducted in a hybrid operating room with fixed angiographic imaging under regional anesthesia (spinal or epidural block with sedation). The patient lay supine, and the ipsilateral common femoral artery was accessed in antegrade fashion using ultrasound guidance and a micropuncture set. A 6–7 French sheath was inserted (the size chosen to accommodate the atherectomy device and balloons). Systemic unfractionated heparin was administered to achieve an activated clotting time of approximately 200–250 s. A 0.014-inch hydrophilic guidewire was then advanced across the target femoropopliteal lesion under roadmap fluoroscopy. In chronic total occlusions, reentry catheters or parallel-wire technique were used as needed.

Atherectomy + Balloon Angioplasty Group

1) Distal Embolic Protection: Before atherectomy, a distal filter device (SpiderFX) was deployed via a contralateral or ipsilateral access into a distal runoff artery (typically the popliteal or tibioperoneal trunk) to capture plaque debris.
2) Atherectomy: A Jetstream rotational atherectomy catheter (Boston Scientific) was advanced over the guidewire to the lesion. The Jetstream system has front-cutting blades and built-in active aspiration, which significantly reduces embolization risk. The cutting diameter was chosen based on vessel size. The operator activated the cutter and incrementally advanced it through the plaque (for example, moving 2 mm forward then 1 mm back) while simultaneously aspirating. Multiple passes were performed, gradually debulking calcium, thrombus, and soft plaque. Care was taken to stop if significant vessel dissection or perforation was noted on fluoroscopy.
3) Debris Removal: The debris-laden filter was periodically inspected. After atherectomy, the filter was carefully collapsed and removed to retrieve embolic material. Visual confirmation of captured fragments ensured adequate plaque removal.
4) Balloon Angioplasty: Immediately following atherectomy, a standard angioplasty balloon (sized 1:1 with the reference vessel diameter) was introduced. High-pressure inflation was performed, typically holding inflation for 2–3 minutes per treatment zone to achieve sufficient luminal gain and limit recoil. In cases of elastic recoil, a non-compliant balloon was used for post-dilation. Completion angiography confirmed brisk blood flow through the treated segment without significant residual stenosis.

Balloon Angioplasty + Stent Placement Group

1) Lesion Preparation: After crossing the lesion with the guidewire, pre-dilation was performed with a plain balloon to create a channel (balloon diameter matched to normal vessel size). Inflation times were around 1–2 minutes, and pressures up to nominal values (8–12 atm) were used. This prepared the lesion for stent placement.
2) Stent Deployment: A self-expanding nitinol stent was selected based on lesion length and vessel diameter. A second-generation drug-eluting stent (DES) was chosen. The stent length covered the entire diseased segment with 5–10 mm margins. Under fluoroscopy, the stent was carefully positioned across the lesion and fully deployed by retracting the delivery sheath.
3) Post-Dilation: Following deployment, a non-compliant balloon (approximately 1 mm larger than the stent diameter) was inflated inside the stent to ensure full expansion and apposition. This post-dilation was held for 2–3 minutes. Completion angiography showed a well-expanded stent with no edge dissection and preserved runoff.

**Postoperative Care and Rehabilitation Protocol**

| **Week** | **Assessment** | **Goals** | **Manual Therapy** | **Supervised Exercises (frequency)** | **Modalities** | **Remarks** |
| --- | --- | --- | --- | --- | --- | --- |
| 1 | Baseline evaluation: assess pain pattern, pulses, ankle-brachial index, 6-minute walk test, treadmill walking test. Screen for comorbidities (heart disease, diabetes, wounds) and contraindications. | Begin safe ambulation; educate on disease and program; establish baseline tolerance. | Gentle soft-tissue work on calves and thighs; passive ankle mobilization; stretching of calf and hamstrings; effleurage massage to legs to promote circulation. | Treadmill walking: 2–5 minute bouts of slow pace to mild leg pain (3/10), alternating with rest until pain subsides. Accumulate ~10–15 minutes walking per session. Frequency: supervised 3 days/week. | Warmth therapy (warm leg packs) before exercise; electrical muscle stimulation (low intensity) or TENS to calves for pain relief; ultrasound to leg muscles as tolerated. | Monitor vitals and claudication closely; allow full rest if chest pain or severe shortness of breath. Encourage tobacco cessation and risk factor control. Provide foot care education. |
| 2 | Re-assess pain-free and maximal walking distance on treadmill; check vitals response to exercise; review gait and posture. | Increase endurance; tolerate longer walking; reinforce education on lifestyle (smoking, diet, blood pressure control). | Continue soft-tissue mobilization and stretching; add gentle joint mobilizations for ankles and feet. | Treadmill walking: extend walking bouts to 5–7 minutes before rest; accumulate 20–25 minutes per session. Add upright cycling or seated marching 5–10 minutes for variety. Frequency: supervised 3 times/week. | Continue warm-up heat; consider contrast bath for calves after exercise; maintain TENS as needed for pain. | Emphasize correct walking technique (heels first); review blood sugar targets if diabetic; encourage patient to walk short distances daily outside sessions. |
| 3 | Check progress: increase in treadmill pain-free interval; measure thigh and calf flexibility; assess leg strength. | Enhance functional walking distance; improve lower limb strength; minimize claudication impact on daily activities. | Soft-tissue massage on lower limbs; add proprioceptive neuromuscular facilitation stretching for calf; myofascial release of iliotibial tract. | Treadmill: walk to moderate claudication (4/10) for ~8–10 minutes, rest, repeat; aim for 30 minutes total (including rests). Introduce light strength: seated leg extensions, heel raises (2 sets of 10) twice weekly. Frequency: supervised 3 times/week. | Add gentle hydrotherapy (warm foot bath) after session if available; therapeutic laser or infrared lamp on leg muscles; maintain TENS/EMS use. | Adjust treadmill speed/incline if patient no longer reaches moderate pain within 8 minutes. Document improvements. Monitor for swelling or pain after exercise; modify if any ulcer appears. |
| 4 | Mid-program check: repeat 6-minute walk or walking impairment questionnaire; re-evaluate ankle pulses and edema. | Achieve 30% gain in pain-free walking time over baseline; improve cardiovascular endurance. | Calf and quadriceps stretching; manual traction for hip joints if stiffness; gentle lumbar mobilization if back tight. | Treadmill: increase continuous walking up to 15 minutes per bout; total ~40 minutes including rest. Cycle ergometer or elliptical 10–15 minutes at low resistance 1–2x/week. Leg strengthening 2–3x/week (lunges, sit-to-stand). | Use pneumatic compression on calves if available to augment venous return. Continue thermal modalities and electrical stimulation. | Address any barriers (arthritis, fear of pain); reinforce home exercise plan. If fatigue or angina, reduce workload and coordinate with physician. |
| 5 | Evaluate symptom changes; check glucose control and blood pressure; inspect feet and nails. | Further extend walking tolerance; transition to moderate pain bouts; maintain joint mobility gains. | Progress manual stretches to PNF techniques for hamstrings/calf; deep tissue massage as needed for muscle soreness. | Treadmill: aim for 20-minute continuous walking at moderate pace (incline 0–2%) to moderate pain; total session time 45 minutes. Continue cycling/swim 15 minutes 1–2x/week. Strength: add resistance (weights/bands) to leg exercises. Frequency: supervised 3 times/week. | Provide contrast hot/cold therapy post-exercise to reduce inflammation; electrical stimulation to gluteal muscles to support gait if needed. | Ensure patient uses symptom-limited intensity; educate on monitoring walking speed and heart rate. Check for signs of claudication flare-up; adjust plan if needed. |
| 6 | Reassess mid-term progress: treadmill distance, 6MWT, walking speed; evaluate ankle ABI. | Reach goal of 50% increase in treadmill walking distance from start; reinforce independent walking habit. | Maintain manual therapy on demand: focus on areas of tightness; mobilize toes/foot if needed. | Treadmill: increase session to 45–50 minutes total. Introduce interval training: walk 5–10 minutes at higher speed/incline to provoke moderate pain, rest, repeat. Add stair stepping or inclines for 5–10 minutes. | Monitor for delayed muscle fatigue; use cryotherapy on legs on off-days if soreness persists. Continue TENS for pain if used. | At week 6, review medications, nutritional intake, and psychological status. Plan for late-phase progression. |
| 7 | Obtain feedback on functional improvements (climbing stairs, walking errands); measure any limb circumference changes. | Consolidate gains; prepare for maintenance phase; strengthen cardiovascular response. | Therapist performs lymphatic drainage if edema present; joint mobilization for stiff hip or knee. | Treadmill: maintain 50-minute sessions; work up to mildly severe claudication (5/10) before rest. Introduce alternating speed (interval) or gradient training. Continue strength 2–3x/week (increase reps/weight gradually). | Evaluate need for new modalities: consider ultrasound deep heating on calf muscles to increase blood flow. Educate on using personal pulse oximeter/HR monitor if available. | Encourage variety: walking in park, treadmill hills. Emphasize wearing proper footwear. Watch for overuse injuries (knee, back). |
| 8 | Interim re-assessment: repeat 6MWT, treadmill test; review ABI and Doppler if indicated. | Ensure at least 60–70% of target walking distance achieved; address residual deficits. | Maintenance: brief manual therapy as needed for recovery; ensure flexibility is sustained. | Treadmill: focus on sustaining longer bouts (15–20 minutes) with moderate pain, minimal rests; total 50+ minutes. Alternative: begin 1 session of outdoor walking of equivalent duration. Strength training focus on endurance (2 sets x 15). | Continue any beneficial modalities; schedule a trial of home NMES if appropriate. | Plan tapering of direct supervision: teach patient to adjust treadmill pace/grade on their own. Discuss long-term goals and lifestyle integration. |
| 9 | Final phase check: evaluate quality-of-life scales (walking impairment questionnaire, ADL status). | Achieve personalized goals (e.g. community ambulation without rest for X minutes); promote independence. | Keep manual therapy minimal; use mostly for persistent tight spots only. | Treadmill: incorporate unstructured walking (dynamic speed changes); aim for 50–60 minutes continual walking to moderate pain. Include 1 session walking on level ground 2 miles if possible. Strength: emphasize functional tasks (sit-stand, step-ups). | Assess need for revascularization referral if progress plateaued despite compliance. Apply modalities as recovery aids between sessions. | Prepare home exercise prescription: encourage 3–5 walks/week. Arrange follow-up assessments (ABI, treadmill) 1–2 months post-program. |
| 10 | Confirm near-goal attainment; review any lingering claudication or lifestyle issues. | Maintain improvements; instill routine exercise habit; prevent relapse of sedentary behavior. | Last manual therapy session focusing on self-care techniques (patient self-massage, stretching education). | Supervised treadmill: final intensive session (e.g. graded test to measure max walking time); reinforce unassisted sessions. Frequency may reduce if patient can self-manage (e.g. 2x/week supervised plus home walks). | Encourage community resources (walking groups, cardiac rehab classes). | Ensure all risk factors are optimally managed (lipids, BP, glycemic control). Discuss psychosocial support if needed to sustain exercise. |
| 11 | Repeat 6MWT and measure improvement from baseline. | Final goal: meet or exceed target distances; ensure patient confidence in continuing program alone. | No routine manual therapy, but coach patient on self-stretching and using foam roller. | Transition to home-based or gym program: at least 3 walking sessions/week for 30–50 minutes, progressing as tolerated. Continue strength and stretching 2–3 times/week. | Advise continued use of heat or TENS at home if helpful. Demonstrate safe progression of exercise intensity. | Provide written exercise plan and warning signs (e.g. chest pain, rest pain) to report. Encourage periodic medical follow-up. |
| 12 | Summative assessment: compare discharge vs baseline (ABI, 6MWT, questionnaires). | Sustain functional gains; quality of life improvement; lifestyle modification reinforcement. | Hand over self-management techniques; full independence in mobility. | Evaluate home exercise adherence. Plan for long-term follow-up in 3–6 months. | Discharge modalities (none in clinic), but advise home use of similar devices if available. | Document outcomes. Celebrate patient’s progress. Emphasize that exercise should continue indefinitely at moderate levels to preserve benefits. |

**Regulation Protocols by physiotherapist**

1) Progression by tolerance: Increase treadmill intensity (speed or incline) once patient can walk ≥10 minutes to moderate claudication without break. If patient can walk beyond 10 min without significant pain, raise workload to provoke moderate pain within 5–10 min as per guidelines. Conversely, if a patient cannot reach target intensity, maintain or reduce load until tolerable improvements occur.
2) Frequency adjustments: Maintain at least three supervised sessions per week; if a patient advances rapidly, allow one session to be unsupervised or remote, but with regular check-ins. If compliance is poor, increase therapist contact or provide additional education/motivation.
3) Alternative exercises: In case of joint pain or foot ulceration, substitute non–weight-bearing exercise (e.g. arm ergometry, seated cycling, or water walking). For patients with diabetic neuropathy or foot wounds, avoid treadmill and use leg ergometry or upper-body aerobic training.
4) Comorbid conditions: Modify intensity for cardiac or pulmonary disease (e.g. target 40–60% heart rate reserve instead of maximal effort). For hypertension or angina, coordinate with the physician and limit exertion (monitor BP and ECG). If severe osteoporosis, reduce impact (use recumbent bike).
5) Contraindications: Do not initiate exercise in presence of critical ischemia (rest pain, ulceration, infection) or unstable cardiovascular conditions. Postpone rehab until wound healing or cardiac clearance. Likewise, acute thrombosis, fracture, or severe COPD exacerbation are contraindications until stabilized.
6) Monitoring and safety: Continuously assess patient’s symptoms, vitals, and claudication level. Use a standard pain scale (0–10) to gauge intensity. Terminate or regress exercise if disproportionate pain, pallor, or ischemic changes occur. Ensure emergency protocols (AED, oxygen) are available during supervised sessions.
7) Lifestyle integration: Encourage continued exercise beyond the 12-week program. Provide strategies (logbooks, goal setting, remote prompts) to sustain adherence. Educate on peripheral vascular risk factors and coordinate with primary care for smoking cessation, diabetes and blood pressure control.

Rehabilitation Protocol as well as its regulation protocols were established at June 2020, the protocol was based on relevant literatures and consented by the Vascular Surgery Branch of Shanghai Medical Association.

**References**

1. Hamburg NM, Balady GJ. Exercise Rehabilitation in Peripheral Artery Disease: Functional Impact and Mechanisms of Benefits. Circulation. 2011;123(1):87-97.
2. Hirsch AT, Haskal ZJ, Hertzer NR, et al. ACC/AHA 2005 practice guidelines for the management of patients with peripheral arterial disease (lower extremity, renal, mesenteric, and abdominal aortic). J Am Coll Cardiol. 2006;47(6):1239-1312.
3. Gerhard-Herman MD, Gornik HL, Barrett C, et al. 2016 AHA/ACC guideline on the management of patients with lower extremity peripheral artery disease. Circulation. 2017;135(12):e726-e779.
4. National Institute for Health and Care Excellence. Peripheral arterial disease: diagnosis and management (NICE guideline CG147). London, NICE; 2012 (updated 2020).
5. Fokkenrood HJP, Bendermacher BL, Lauret GJ, et al. Supervised exercise therapy versus non-supervised exercise therapy for intermittent claudication (Cochrane Review). Cochrane Database Syst Rev. 2013;(8):CD005263.

**Supplementary Table 1 Baseline characteristics of the "Atherectomy + Balloon Angioplasty" Group and the "Balloon Angioplasty + Stent Placement" Group (per-protocol population)**

| **PP** | **Characteristic** | **AB group (N=142)** | **BS Group (N=145)** | **P value** |
| --- | --- | --- | --- | --- |
| **Baseline Characteristics** | Male Gender (no. [%]) | 78 (54.93) | 78 (53.79) | 0.847 |
|  | Age (yrs) | 61 (5.01) | 60 (5.64) | 0.925 |
|  | BMI (kg/m²) | 23 (2.81) | 23 (3.12) | 0.663 |
|  | Occupation |  |  | 0.690 |
|  | Manual worker | 118 (83.10) | 123 (84.83) |  |
|  | Non-manual worker | 24 (16.90) | 22 (15.17) |  |
|  | Education level |  |  | 0.891 |
|  | Lower than high school | 54 (38.03) | 54 (37.24) |  |
|  | Equal/higher to high school | 88 (61.97) | 91 (62.76) |  |
|  | Insurance type |  |  | 0.986 |
|  | Government | 104 (73.24) | 105 (72.41) |  |
|  | Commercial | 3 (2.11) | 3 (2.07) |  |
|  | Self | 35 (24.65) | 37 (25.52) |  |
|  | Current Smoker | 49 (34.51) | 48 (33.10) | 0.802 |
|  | Current Alcohol use | 50 (35.21) | 54 (37.24) | 0.721 |
|  | Paracetamol and NSAIDs | 7 (4.38) | 5 (3.13) | 0.588 |
| **Medical History** | Coronary Artery Disease (CAD) | 5 (3.52) | 5 (3.45) | 0.973 |
|  | History of stroke or TIA, or known carotid artery stenosis. | 4 (2.82) | 5 (3.45) | 0.759 |
|  | History of congestive heart failure (reduced ejection fraction or HFpEF) | 4 (2.82) | 3 (2.07) | 0.681 |
|  | Presence of COPD or asthma requiring ongoing treatment. | 3 (2.11) | 5 (3.45) | 0.492 |
|  | Chronic Kidney Disease | 3 (2.11) | 4 (2.76) | 0.723 |
|  | Antiplatelet Therapy use | 121 (85.21) | 121 (83.45) | 0.681 |
|  | Statin Therapy use | 141 (99.30) | 145 (100.00) | 0.985 |
|  | PAD Symptom Duration (months) | 72 (7.85) | 72 (7.51) | 0.854 |
| **Limb-specific Data and History** | Affected Limb Laterality |  |  | 0.971 |
|  | Left | 80 (56.34) | 82 (56.55) |  |
|  | Right | 62 (43.66) | 63 (43.45) |  |
|  | Lesion Location (Arterial Segment) |  |  | 0.060 |
|  | Aorto-iliac | 2 (1.41) | 9 (6.21) |  |
|  | Femoropopliteal | 55 (38.73) | 45 (31.03) |  |
|  | Infra-popliteal (tibial/pedal) | 85 (59.86) | 91 (62.76) |  |
|  | Lesion Length |  |  | 0.196 |
|  | focal ≤1 cm | 30 (21.13) | 26 (17.93) |  |
|  | short >1–<5 cm | 87 (61.27) | 97 (66.90) |  |
|  | intermediate 5–<15 cm | 24 (16.90) | 17 (11.72) |  |
|  | long ≥15 cm | 1 (0.70) | 5 (3.45) |  |
|  | Degree of Stenosis |  |  | 0.284 |
|  | mild (<50%) | 45 (31.69) | 48 (33.10) |  |
|  | moderate (50–69%) | 92 (64.79) | 86 (59.31) |  |
|  | severe (70–99%) | 5 (3.52) | 11 (7.59) |  |
|  | Multi-level disease | 14 (9.86) | 21 (14.48) | 0.231 |
| **Function** | Ankle-Brachial Index (ABI) | 0.73 (0.06) | 0.71 (0.06) | 0.053 |
|  | 6-minute walk test (6MWT) (m) | 242 (49.86) | 242 (57.19) | 0.998 |
|  | Rutherford classification for Intermittent Claudication |  |  | 0.480 |
|  | Mild | 9 (6.34) | 11 (7.59) |  |
|  | Moderate | 131 (92.25) | 129 (88.97) |  |
|  | Severe | 2 (1.41) | 5 (3.45) |  |
|  | Peripheral Artery Questionnaire (PAQ) | 53 (6.05) | 52 (5.94) | 0.558 |

AB: Atherectomy + Balloon Angioplasty; BS: Balloon Angioplasty + Stent Placement; PP: per-protocol; BMI: body mass index; NSAIDs: Non-Steroidal Anti-Inflammatory Drugs; HFpEF: Heart Failure with Preserved Ejection Fraction; TIA: Transient Ischemic Attack; COPD: Chronic Obstructive Pulmonary Disease; PAD: Peripheral Arterial Disease.

* Values were reported as mean (standard deviation) for age, BMI and all variables of function (ABI, 6MWT, PAQ), others were reported as number (percentage).

**Supplementary Table 2 Unadjusted Changes in outcomes of the "Atherectomy + Balloon Angioplasty" Group and the "Balloon Angioplasty + Stent Placement" Group at month 3, 6 and 12 (per-protocol population)**

| **Outcomes*** | **3-month** |  |  | **6-month** |  |  | **12-month** |  |  |
| --- | --- | --- | --- | --- | --- | --- | --- | --- | --- |
|  | **AB group (N=142)** | **BS Group (N=145)** | **P value** | **AB group (N=142)** | **BS Group (N=145)** | **P value** | **AB group (N=142)** | **BS Group (N=145)** | **P value** |
| Primary Patency | 141 (99.30) | 144 (99.31) | 0.988 | 139 (97.89) | 143 (98.62) | 0.635 | 139 (97.89) | 142 (97.93) | 0.979 |
| Re-occlusion in last 3 months verified by Duplex Ultrasound | 1 (0.70) | 1 (0.69) | 0.988 | 2 (1.41) | 1 (0.69) | 0.549 | 0 (0.00) | 1 (0.69) | 1.000 |
| Ankle-Brachial Index change from baseline | 0.15 (0.09) | 0.16 (0.08) | 0.169 | 0.15 (0.10) | 0.17 (0.10) | 0.271 | 0.17 (0.10) | 0.17 (0.10) | 0.723 |
| 6-minute walk test (6MWT) (m) | 104.32 (60.65) | 106.30 (70.79) | 0.799 | 113.92 (67.69) | 113.76 (76.65) | 0.985 | 122.21 (70.62) | 119.55 (79.23) | 0.764 |
| Rutherford classification |  |  | 0.447 |  |  | 0.999 |  |  | 0.879 |
| Mild | 129 (90.85) | 125 (86.21) |  | 132 (92.96) | 135 (93.10) |  | 135 (95.07) | 136 (93.79) |  |
| Moderate | 12 (8.45) | 19 (13.10) |  | 8 (5.63) | 8 (5.52) |  | 6 (4.23) | 8 (5.52) |  |
| Severe | 1 (0.70) | 1 (0.69) |  | 2 (1.41) | 2 (1.38) |  | 1 (0.70) | 1 (0.69) |  |
| Peripheral Artery Questionnaire (PAQ) | 17.27 (7.89) | 18.08 (8.42) | 0.398 | 22.89 (9.10) | 22.89 (7.37) | 0.998 | 25.48 (8.76) | 26.12 (8.89) | 0.541 |
| Mortality and Major Adverse Cardiovascular Events (MACE) | 2 (1.41) | 2 (1.38) | 0.983 | 0 (0.00) | 0 (0.00) | 1.000 | 0 (0.00) | 0 (0.00) | 1.000 |

AB: Atherectomy + Balloon Angioplasty; BS: Balloon Angioplasty + Stent Placement.

*Primary Patency was assessed by duplex ultrasound patency is maintained if the duplex shows <50% diameter restenosis;

Mortality and Major Adverse Cardiovascular Events: events related to myocardial infarction, stroke and cardiovascular death;

Values represent the mean change from baseline for each group, reported as mean (standard deviation), with P values comparing between-group differences at each follow-up point. All outcome measures were unadjusted.

**Supplementary Table 3 Costs of the "Atherectomy + Balloon Angioplasty" Group and the "Balloon Angioplasty + Stent Placement" Group at month 12 (per-protocol population)**

| **PP** | **AB group (N=160)** | **BS Group (N=160)** | **P value** |
| --- | --- | --- | --- |
| **Direct medical cost** (CNY) |  |  |  |
| Hospital stays cost (CNY) | 6969 (969.74) | 6949 (1013.26) | 0.865 |
| Primary care cost (CNY) | 2511 (463.43) | 2509 (515.04) | 0.968 |
| Secondary care cost (CNY) | 2937 (3117.72) | 2977 (3207.43) | 0.915 |
| Physical therapist cost (CNY) | 10318 (1981.99) | 10103 (2031.43) | 0.365 |
| Implantation/Device/Medication (CNY) | 27587 (2455.82) | 54546 (4595.10) | 0.000 |
| **Direct non-medical cost** (CNY) |  |  |  |
| Transportation cost (CNY) | 1987 (306.30) | 1990 (308.67) | 0.935 |
| Nutrition cost (CNY) | 4455 (527.11) | 4486 (442.52) | 0.581 |
| **Opportunity Cost** (CNY) |  |  |  |
| Lost wages for patients (CNY) | 32765 (17542.88) | 32215 (16473.24) | 0.784 |
| Lost wages for families (CNY) | 3905 (4547.39) | 3950 (4245.12) | 0.930 |
| **Total cost** (CNY) | 93434 (18719.88) | 119726 (18460.70) | 0.000 |

PP: per-protocol; CNY: Chinese Yuan; AB: Atherectomy + Balloon Angioplasty; BS: Balloon Angioplasty + Stent Placement.

All relevant costs were captured from a societal perspective. This included direct medical costs (Hospital stay, primary and secondary care, rehabilitation and physical therapy costs and medication costs), direct non-medical costs related to care (transportation for medical visits and any specialized nutritional support during recovery), and indirect costs due to productivity loss (lost wages for patients during recovery or disability, and lost income for family members/caregivers, if applicable). Costs were obtained from hospital billing records and patient self-reports where needed (wage losses). All costs are reported in 2024 Chinese Yuan, as 2024 was the end of the enrollment period; costs incurred in earlier years were adjusted to 2024 price levels using the consumer price index. Costs and outcomes occurring beyond the initial year were discounted at an annual rate of 3% to reflect time preference, consistent with standard practice in health economic evaluations.

**Supplementary Table 4 Adverse events and serious adverse events (intention-to-treat population)**

| **AE** | **AB group (N=160)** | **BS Group (N=160)** |
| --- | --- | --- |
| Patients with adverse events (no. [%]) ^#^ | 5 (3.1) | 3 (1.9) |
| Events related to surgery (no.) |  |  |
| Access-site bleeding/hematoma | 1 | 0 |
| Superficial infection treated with conservative method | 1 | 0 |
| Pseudoaneurysm or arteriovenous fistula | 0 | 1 |
| Arterial dissection | 0 |  |
| Distal embolization | 0 | 1 |
| Vessel perforation | 1 | 0 |
| Events unrelated to study (no.) |  |  |
| Ankle sprain | 1 | 0 |
| Low back pain | 1 | 0 |
| Anxiety | 0 | 1 |
| **SAE*** |  |  |
| Patients with serious adverse eve nts (no. [%]) | 1 (0.6) | 2 (1.2) |
| Events related to surgery (no.) |  |  |
| Major limb loss (amputation) | 0 | 0 |
| Major bleeding | 0 | 0 |
| Death (perioperative mortality) | 0 | 0 |
| Events unrelated to study (no.) |  |  |
| Hip fracture due to fall | 1 | 0 |
| Unplanned surgery | 0 | 2 |

AE: adverse events; SAE: serious adverse events; AB: Atherectomy + Balloon Angioplasty; BS: Balloon Angioplasty + Stent Placement.

Values of AEs and SAEs were reported as number (percentage) in intent-to-treat population

*Patients with serious adverse events were automatically withdrawn from the study

Access-site bleeding/hematoma is defined as bleeding at the vascular access puncture site resulting in a hematoma. This includes any bleeding requiring extended compression, thrombin injection, or blood transfusion at the catheter entry site.

Pseudoaneurysm or arteriovenous fistula is defined as injury to the arterial wall at the access site causing either a pseudoaneurysm or an arteriovenous (AV) fistula. A pseudoaneurysm (“false aneurysm”) occurs when an arterial wall is injured and blood leaks into surrounding tissue, forming a contained pulsatile hematoma.

Arterial dissection is defined as a tear of the artery’s intimal layer created during the procedure, allowing blood to enter and split the vessel wall.

Distal embolization is defined as detachment of atherosclerotic plaque, thrombus, or debris during the intervention that travels downstream and lodges in smaller distal arteries.

Vessel perforation is defined as an unintended full-thickness puncture or rupture of the arterial wall during the procedure.

Major limb loss (amputation) is defined as surgical removal of the lower limb at or above the ankle. By convention, a “major amputation” refers to any leg amputation above the ankle (e.g. below-knee or above-knee amputation).

Major bleeding is defined as severe hemorrhage meeting standard criteria for life-threatening bleeding. This includes fatal bleeding or bleeding into a critical site (e.g. intracranial, intraocular, retroperitoneal), or bleeding causing a hemoglobin drop ≥2.0 g/dL (20 g/L) or requiring transfusion of ≥2 units of blood.

Death is defined death of the patient from any cause during surgery.

**Supplementary Table 5 Standardized mean differences of baseline covariates before and after propensity score matching**

|  | **Characteristic** | **SMD before matching** | **SMD after matching** | **AB Group matched (N=160)** | **BS Group matched (N=160)** |
| --- | --- | --- | --- | --- | --- |
| **Baseline Characteristics** | Male Gender | 0.096 | 0.025 | 91 (56.88) | 89 (55.63) |
|  | Age (yrs) | 0.355 | 0.019 | 60 (4.99) | 60 (5.54) |
|  | BMI (kg/m^2) | 0.324 | 0.034 | 23 (2.85) | 23 (3.09) |
|  | Manual worker | 0.118 | 0.017 | 135 (84.38) | 136 (85.00) |
|  | Lower than high school | 0.159 | 0.026 | 63 (39.38) | 61 (38.13) |
|  | Government insurance | 0.113 | 0.014 | 120 (75.00) | 119 (74.38) |
|  | Current Smoker | 0.081 | 0.026 | 56 (35.00) | 54 (33.75) |
|  | Current Alcohol use | 0.020 | 0.026 | 56 (35.00) | 58 (36.25) |
|  | Paracetamol and NSAIDs | 0.074 | 0.067 | 7 (4.38) | 5 (3.13) |
| **Medical History** | Coronary Artery Disease | 0.154 | 0.067 | 7 (4.38) | 5 (3.13) |
|  | Stroke/TIA or carotid stenosis | 0.136 | 0.038 | 4 (2.50) | 5 (3.13) |
|  | Congestive heart failure | 0.104 | 0.043 | 4 (2.50) | 3 (1.88) |
|  | COPD or asthma | 0.119 | 0.034 | 5 (3.13) | 6 (3.75) |
|  | Chronic Kidney Disease | 0.134 | 0.078 | 3 (1.88) | 5 (3.13) |
|  | Antiplatelet Therapy | 0.082 | 0.052 | 136 (85.00) | 133 (83.13) |
|  | Statin Therapy | 0.122 | 0.000 | 158 (98.75) | 160 (100.00) |
|  | PAD Symptom Duration | 0.231 | 0.000 | 72 (7.69) | 72 (7.48) |
| **Limb-specific Data** | Left laterality | 0.132 | 0.101 | 87 (54.38) | 95 (59.38) |
|  | Femoropopliteal location | 0.210 | 0.077 | 64 (40.00) | 58 (36.25) |
|  | Severe stenosis (70-99%) | 0.471 | 0.078 | 8 (5.00) | 11 (6.88) |
|  | Multi-level disease | 0.272 | 0.091 | 19 (11.88) | 24 (15.00) |
| **Function** | Ankle-Brachial Index | 0.707 | 0.330 | 0.73 (0.06) | 0.71 (0.06) |
|  | 6-minute walk test (m) | 0.405 | 0.019 | 241 (50.55) | 240 (56.34) |
|  | Rutherford severe | 0.609 | 0.135 | 2 (1.25) | 5 (3.13) |
|  | Peripheral Artery Questionnaire | 0.760 | 0.022 | 53 (5.90) | 52 (6.04) |

SMD: standardized mean difference; AB: Atherectomy + Balloon Angioplasty; BS: Balloon Angioplasty + Stent Placement; BMI: body mass index; NSAIDs: Non-Steroidal Anti-Inflammatory Drugs; TIA: Transient Ischemic Attack; COPD: Chronic Obstructive Pulmonary Disease; PAD: Peripheral Arterial Disease. An SMD below 0.10 indicates negligible imbalance. Before matching, several covariates exceeded this threshold; after matching, all covariates fell below 0.10 except baseline ABI (SMD 0.33), which was included as a covariate in all adjusted models. The corresponding Love plot is shown in Supplementary Figure 1.

**Supplementary Table 6 Exploratory subgroup analysis of 12-month primary patency by lesion location (intention-to-treat)**

| **Lesion location** | **AB group patency [n/N (%)]** | **BS Group patency [n/N (%)]** | **P value** | **Risk difference (95% CI)** |
| --- | --- | --- | --- | --- |
| Femoropopliteal | 60/61 (98.36) | 54/55 (98.18) | 1.000 | 0.2% (-4.7% to 5.1%) |
| Infra-popliteal | 89/91 (97.80) | 96/98 (97.96) | 1.000 | -0.2% (-4.2% to 3.9%) |

AB: Atherectomy + Balloon Angioplasty; BS: Balloon Angioplasty + Stent Placement; CI: confidence interval. Denominators reflect the number of patients with an available 12-month patency assessment within each lesion-location subgroup. Between-group comparisons used Fisher’s exact test. These subgroup analyses were prespecified as exploratory and are underpowered; they should not be interpreted as definitive lesion-specific estimates. Femoropopliteal and infrapopliteal lesions differ in treatment strategy, expected patency, indications for stenting, and reintervention risk, and the overall study findings should not be generalized to lesion-specific recommendations.

**Supplementary Figure 1**

Love plot of standardized mean differences before and after propensity score matching. The plot displays the absolute standardized mean difference for each baseline covariate before matching (open circles) and after matching (filled circles); the vertical reference line marks the 0.10 threshold for negligible imbalance. After matching, all covariates fell below 0.10 except baseline ankle-brachial index.
